# Nanoparticle enhanced combination therapy for stem-like progenitors defined by single-cell transcriptomics in chemotherapy-resistant osteosarcoma

**DOI:** 10.1038/s41392-020-00248-x

**Published:** 2020-09-25

**Authors:** Li Wang, Xiaojia Huang, Xinru You, Tianqi Yi, Bing Lu, Jiali Liu, Guohao Lu, Minglin Ma, Changye Zou, Jun Wu, Wei Zhao

**Affiliations:** 1 RNA Biomedical Institute, Sun Yat-Sen Memorial Hospital, Sun Yat-Sen University, Guangzhou, 510120 China; 2 Key Laboratory of Stem Cells and Tissue Engineering (Sun Yat-Sen University), Ministry of Education, Guangzhou, China; 3 School of Biomedical Engineering, Sun Yat-sen University, Guangzhou, 510006 China; 4 Department of Biological and Environmental Engineering, Cornell University, Ithaca, NY 14853 USA; 5 Musculoskeletal Oncology Center, The First Affiliated Hospital of Sun Yat-Sen University, Guangzhou, 510080 China; 6 Research Institute of Sun Yat-Sen University in Shenzhen, Shenzhen, 518057 China

**Keywords:** Bone cancer, Cancer stem cells, Drug delivery

## Abstract

The adaptation of osteosarcoma cells to therapeutic pressure impedes the efficacy of chemotherapy for osteosarcoma. However, the characteristics and cellular organization of therapy-resistant cells in osteosarcoma tumors remain elusive. Here, we utilized single-cell transcriptomics to systematically map the cell-type-specific gene expression in a chemotherapy-resistant osteosarcoma tumor. Our data demonstrated the VEGFR2-JMJD3-abundant subsets as quiescent stem-like cells, thereby establishing the hierarchy of therapy-resistant actively cycling progenitor pools (JMJD3-abundant) in osteosarcoma. VEGFR2 inhibitor and JMJD3 inhibitor synergistically impeded osteosarcoma cell propagation and tumor growth. Although osteosarcoma cells are predisposed to apoptosis induced by the synergistic therapy through activation of the CHOP pro-apoptotic factor via the endoplasmic reticulum (ER) stress, the stem-like/progenitor cells exhibit an adaptive response, leading to their survival. Reduction in cellular glutathione levels in stem-like/progenitor cells caused by the treatment with a glutathione synthesis inhibitor increases ER stress-induced apoptosis. Importantly, the marked therapeutic improvement of synergistic therapy against stem-like/progenitor cells was achieved by using glutathione-scavenging nanoparticles, which can load and release the drug pair effectively. Overall, our study provides a framework for understanding glutathione signaling as one of the therapeutic vulnerabilities of stem-like/progenitor cells. Broadly, these findings revealed a promising arsenal by encapsulating glutathione-scavenging nanoparticles with co-targeting VEGFR2 and JMJD3 to eradicate chemotherapy-resistant osteosarcoma.

## Introduction

Osteosarcoma, the most common primary malignant bone tumor in children and adolescents, is frequently resistant to standard therapies.^1^ There is an urgent need for improving the efficacy of treatment regimens and identifying new therapeutic targets in this disease.^2^ A small subpopulation of osteosarcoma cells that bear self-renewal characteristics of stem cells are responsible for the tumor’s drug resistance and high metastatic potential.^3,4^ Therefore, it is important to have an in-depth understanding of the properties and molecular mechanisms of these chemotherapy-resistant cells.

Recent genetic advances have characterized the genomic landscape of osteosarcoma, including marked inter-tumoral/intrinsic heterogeneity and rare recurrent driver mutations.^5^ Preclinical studies revealed biological pathways implicated in tumorigenesis and therapy resistance, such as NF-kB, PI3K/mTOR, and WNT/β-catenin.^6–9^ However, in preclinical studies, cell culture of primary osteosarcoma cells or cell lines undergo extensive genetic changes and lose their phenotypic heterogeneity; thus, the conclusions from studies made with these cells are different from those of the primary tumors. Single-cell transcriptomics technologies can reveal cellular heterogeneity within a cell population. Therefore, the single-cell transcriptomics data sets obtained from therapy-resistant tumors enable a deeper understanding of the critical biological processes and the mechanisms that drive drug resistance.

To date, the cell-type of therapy-resistant cells in osteosarcoma tumors is not clearly defined. A hypothesis that explains the chemotherapy resistance of tumors is the existence of a subpopulation of cancer cells with stem/progenitor properties (so-called cancer stem cells) in osteosarcoma.^10,11^ Cancer stem cells possess several characteristics that contribute to the development of drug resistance, including slow cell cycle, rapid response to DNA damage, resistance to oxidative stress, and the ability to mediate the efflux of cytotoxic agents.^12^ In osteosarcoma, previous studies showed that side population (SP) of cells, exhibiting increased sphere-forming capabilities and higher tumorigenicity, may be enriched in the cancer stem cells population.^13^ Moreover, CD117 and Stro-1 double-positive osteosarcoma cells showed high invasiveness, drug resistance, and a potential for self-renewal and metastasis.^14^ In addition to surface markers, there is an increasing interest in the key signaling pathways in osteosarcoma cancer stem cells for the development of new therapies that aim to eliminate these cells.^15^ Further in-depth understanding of the maintenance and hierarchy of these so-called cancer stem cells in osteosarcoma will improve osteosarcoma prognosis and treatment.

Heterogeneity leads to a variability in the sensitivity of tumor cells to therapeutic drugs. Several studies have reported that metabolism reprograming (e.g., oxygen metabolism) contributes to drug resistance.^16^ Glutathione (GSH) and GSH-related enzymes constitute an important antioxidant defense system that protects cells from chemotherapy attacks. The GSH system is coupled with intracellular redox dynamic balances through the exchange of free thiol/disulfide bonds, thus maintaining energy metabolism, homeostasis, and regulating drug responsiveness.^17^ It has been found that TMZ-resistant glioma cells displayed lower levels of endogenous reactive oxygen species and higher levels of intracellular GSH than sensitive cells.^18^ Minimal attention has been paid to the role of the GSH redox system in osteosarcoma drug resistance.

**Scheme 1:**
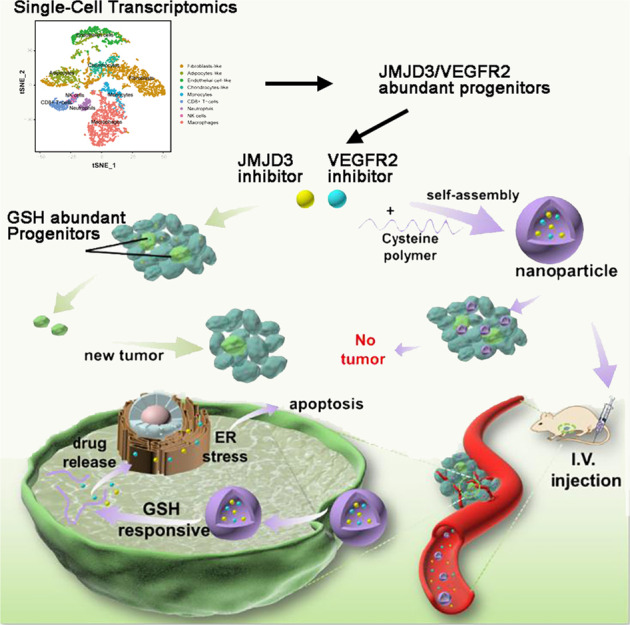
Schematic illustration of GSH-scavenging nanoparticles for synergistic suppression of chemotherapy-resistant osteosarcoma. Single-cell transcriptomics indicate VEGFR2-JMJD3-abundant cells in the stem-like/progenitor populations in osteosarcoma. NP_J4+Apa_ could effectively load and release the Apa (VEGFR2 inhibitor) and J4 (JMJD3 inhibitor), increase endoplasmic reticulum stress-induced apoptosis, and exhibit significant potency against osteosarcoma stem-like/progenitor cell-derived tumors

In this study, we sought to explore the cell heterogeneity in therapy-resistant stem-like/progenitor cells during osteosarcoma tumorigenesis at the single-cell level. We revealed the VEGFR2-JMJD3-abundant subsets as the slow-cycling stem-like cells establish a hierarchy of therapy-resistant actively cycling JMJD3-abundant progenitors. Moreover, we found that co-targeting VEGFR2 and JMJD3 by encapsulating GSH-scavenging nanoparticles (NPs) could effectively eradicate chemotherapy-resistant osteosarcoma stem-like/progenitor cell-derived tumors (Scheme 1).

## Results

### Single-cell transcriptomics reveal stem-like/progenitor populations in chemo-resistant osteosarcoma

**Fig. 1:**
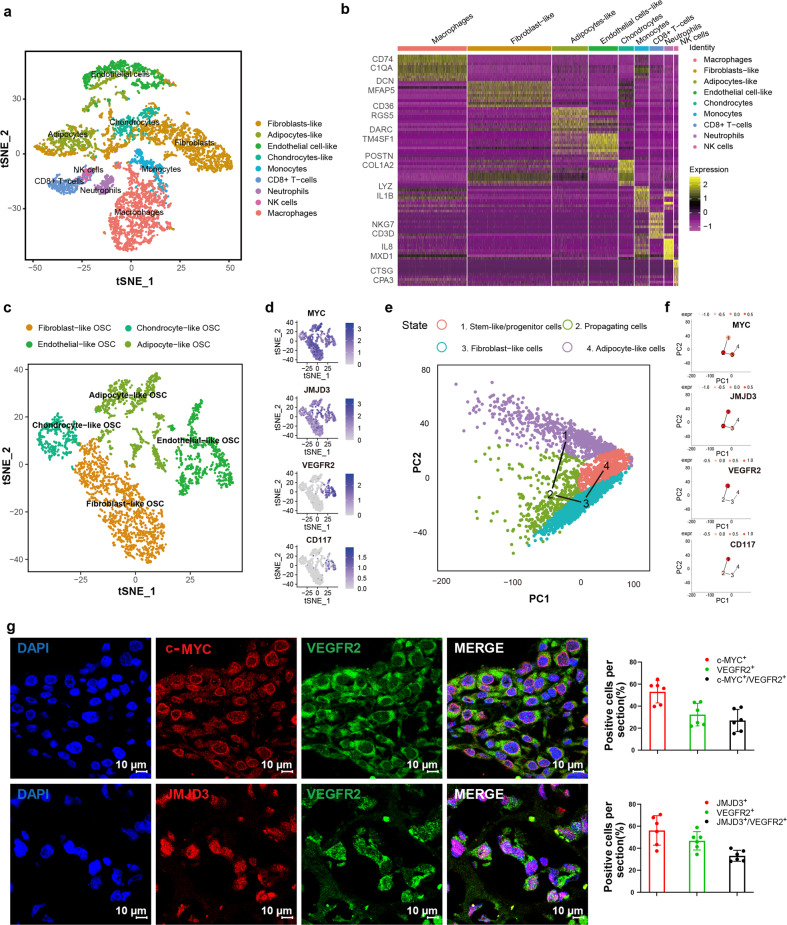
scRNA-seq transcriptome illustrates the heterogeneity of osteosarcoma in a chemotherapy-resistant patient. **a** t-SNE and clustering analysis of single-cell transcriptome data from osteosarcoma (*n* = 7177). **b** Heatmap showing the top 10 differentially expressed genes (DEGs) between clusters. Each row represents a gene, and each column represents a single cell. **c** Re-clustering and focused analysis of the osteosarcoma cells (OSC) (*n* = 3168). **d** t-SNE plots of expression of osteosarcoma-associated markers. **e** Pseudo-time (via TSCAN) ordering of distinct populations within therapy-resistant osteosarcoma. **f** TSCAN plots showing the expression of JMJD3 and VEGFR2 in stem-like/progenitor populations. Expr expression level. **g** Immunofluorescence staining showed that MYC^+^/JMJD3^+^ or JMJD3^+^/VEGFR2^+^ double-positive cell subsets were found in the chemo-resistant osteosarcoma samples (*n* = 6). The scale length of each image is 10 μm

We performed a single-cell RNA sequencing (scRNA-seq) in osteosarcoma tumor cells without prior cell-type selection using a specimen obtained from a chemotherapy-resistant patient (Supplementary Fig. 1a). Pathological examination confirmed that the tumor was an invasive osteosarcoma. Nine transcriptionally distinct cell clusters were identified using t-SNE analysis (Fig. 1a). Gene expression profiling of 7177 cells reflected the characteristics of the tumor and the surrounding microenvironment (Fig. 1b) and indicated extensive (38.92%) immune cell infiltration. According to the cell-specific marker expression (Supplementary Fig. 1b), these immune cell groups may represent macrophages (25.18%), monocytes (5.15%), neutrophils (3.25%), natural killer (NK) cells (1.62%), and CD8^+^ T cells (5.13%).

We removed the above-mentioned immune cells from the mixed population. Unbiased clustering was used to subdivide the rest of the cell populations into fibroblast-like, chondrocyte-like, endothelial-like, and adipocyte-like osteosarcoma cells (Fig. 1c, Supplementary Fig. 1c). Consistent with previous data, the endothelial marker *DARC*, chondrocyte marker *POSTN*, fibroblast marker *MFAP5*, and adipocyte marker *CD36* were specifically expressed in the corresponding populations (Supplementary Fig. 1d). Consensus profiles indicated that the endothelial-like cells shared common carcinoma markers such as *MYC*, the epigenetic regulator *JMJD3*, the angiogenesis marker *VEGFR2*, and stem cell markers such as *CD117* (Fig. 1d). Violin plots showed similar expression patterns of *MYC*, *JMJD3*, and *VEGFR2* in endothelial-like populations (subpopulations 4, 6, and 9) (Supplementary Fig. 1e).

We conducted unsupervised pseudo-time analysis using TSCAN (Fig. 1e) and revealed a developmental trajectory from stem-like/progenitor (endothelial-like) cells expressing *CD117* to propagating (chondrocyte-like) cells that express *COL8A1* and representative markers of each cell cycle phase (Supplementary Fig. 1f). Strikingly, *MYC* and *JMJD3* were enriched in the cluster of the stem-like/progenitor and propagating cells. The distribution pattern of *VEGFR2* coincided with that of *CD117* and was enriched in the stem-like/progenitor cells (Fig. 1f). Furthermore, JMJD3^+^/VEGFR2^+^ cell subset was found in more chemotherapy-resistant osteosarcoma tumors (*n* = 6), and the proportion of JMJD3^+^/VEGFR2^+^ double-positive cells was more than 20% in these samples (Fig. 1g). We also performed another pseudo-time analysis strategy using the Monocle package, which generated results consistent with those obtained in the TSCAN analyses, confirming that *VEGFR2*^+^ endothelial-like cells (subpopulation 4, 6, and 9) serve as roots for differentiation (Supplementary Fig. 1g). They either differentiated toward chondrocyte-like (subpopulation 3, 5, and 8) or fibroblast-like (subpopulation 0, 1, and 2) cell lineages (Supplementary Fig. 1h).

### Inhibition of JMJD3 and VEGFR2 synergistically suppresses the proliferation of osteosarcoma cells

**Fig. 2:**
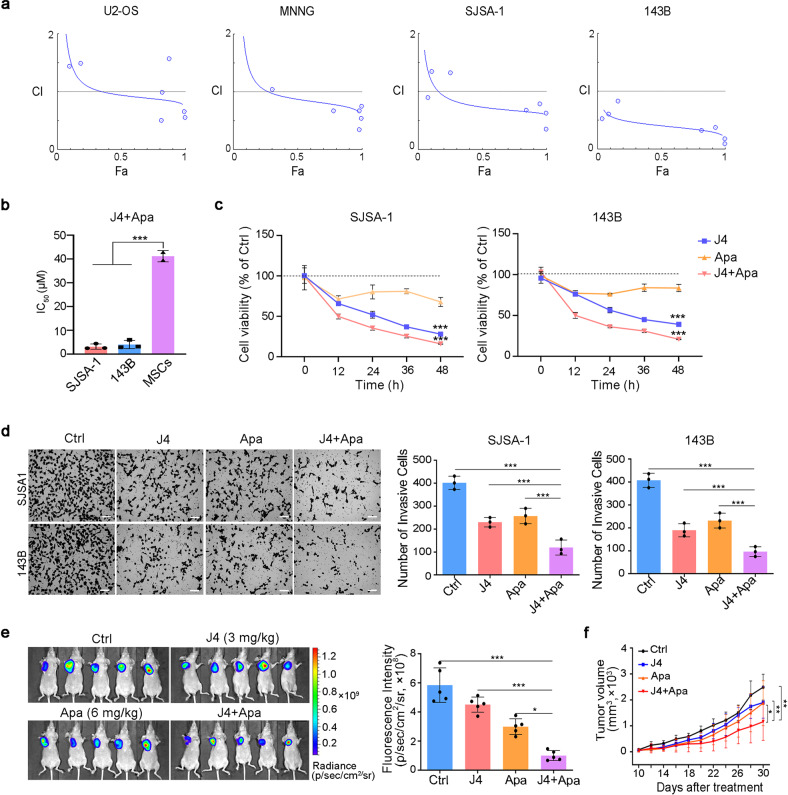
J4 synergizes with Apa in the suppression of osteosarcoma tumor growth. **a** Analysis of the synergism between J4 and Apa. The corresponding combination index (CI) vs. fraction affected (Fa) plots of J4 and Apa under different drug concentration conditions in osteosarcoma cell lines. **b** IC_50_ values of SJSA-1 and 143B (non-stem-like) and MSCs treated with J4+Apa for 48 h. **c** The cell viability inhibition curve of J4 or/and Apa in SJSA-1 or 143B cells (non-stem-like). **d** The non-stem-like cells were treated with different formulations for 24 h in Matrigel matrix-coated invasion chambers. Cells that reached the bottom of the membranes were photographed (scale bar = 200 μm). **e**, **f** SJSA-1 cells (non-stem-like) were subcutaneously injected into the right hind leg to establish osteosarcoma xenograft model (**e**). The tumor-bearing nude mice were administered intravenously (i.v.) with different therapeutic formulations. The representative images at day 30 are presented as **f** tumor growth curves of different formulations in SJSA-1 (non-stem-like) tumor-bearing mice. *P* values were calculated using Student’s *t*-test (***P < 0.001, **P < 0.01, or *P < 0.05)

To inform the clinical translation of the findings of single-cell transcriptomics, we prioritized the targets of JMJD3 and VEGFR2, for which the small-molecule inhibitors were available. We found that different concentrations of the JMJD3 inhibitor GSK-J4 (J4) and VEGFR2 inhibitor Apatinib (Apa) combinations had a synergistic effect in suppressing osteosarcoma cells, particularly at higher concentrations (Fig. 2a). Importantly, this drug pair had no toxic effect on mesenchymal stem cells (Fig. 2b). Further experiments with the two high-risk and metastatic osteosarcoma cell lines (SJSA-1 and 143B) were performed using 5 μM of J4 and/or 10 μM of Apa, as the drug combination at these concentrations had the lowest combination index, while reflecting the highest level of synergy. Both cell lines treated with this drug combination exhibited a more significant time-dependent decrease in cell viability compared to that in cells treated with the single drug alone (Fig. 2c).

We also assessed the effects of J4 and Apa co-treatment on the cell cycle in SJSA-1 and 143B cells. As shown in Supplementary Fig. 2a, J4 and Apa co-administration induced cell cycle blockade in the S phase with reduction of G2/M populations in both cell lines. Metastasis is one of the main causes of osteosarcoma therapy failure. To examine the synergistic effect of J4 and Apa on cell invasion and migration, transwell and wound-healing assays were performed using the SJSA-1 and 143B cell lines. The J4 and Apa combination treatment significantly and synergistically inhibited cell invasion (Fig. 2d) and migration (Supplementary Fig. 2b) capabilities of both cell lines compared with the single-agent treatments.

We further tested the antitumor activity of the drug combination in the SJSA-1 xenograft model. Fluorescent images of the tumors treated with an intravenous infusion of combined 3 mg/kg J4 and 6 mg/kg Apa were compared with those of tumors treated with the respective single drugs alone. According to the fluorescence intensity, the combination treatment showed significantly stronger (*P* < 0.01) tumor growth inhibition than the single drug after six rounds of treatments (from the 10th to the 30th day of tumor growth) (Fig. 2e, f).

### Inhibition of JMJD3 and VEGFR2 enhances the ER stress-induced apoptosis of osteosarcoma cells

**Fig. 3:**
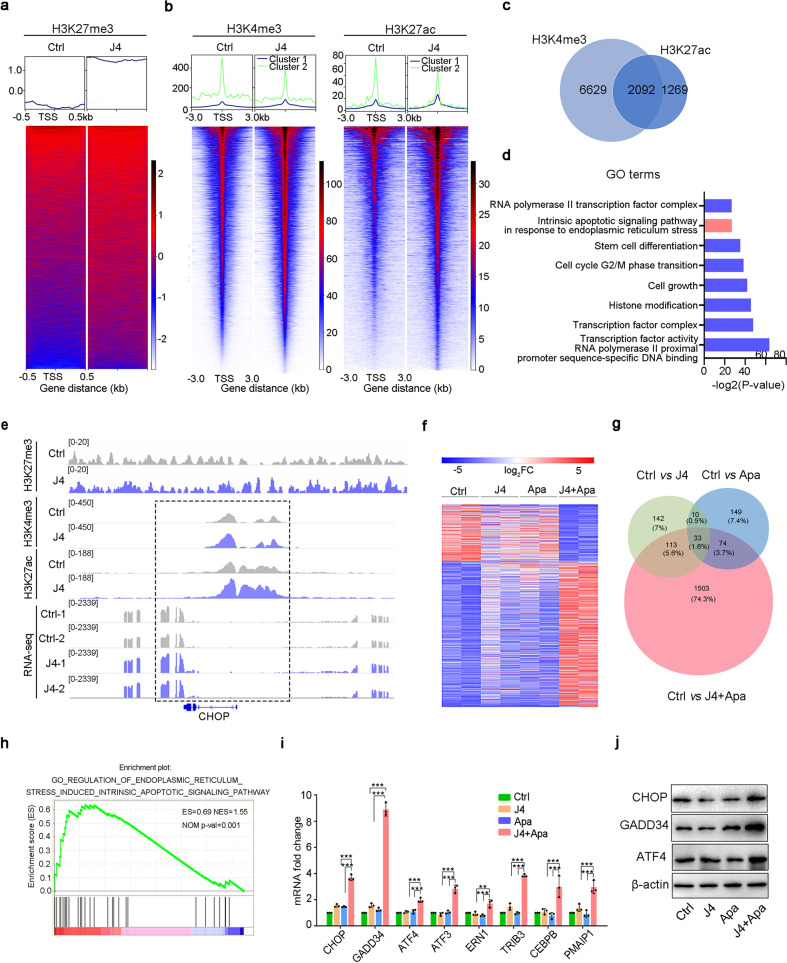
Mechanism of synergistic effect of the dual-drug combination. **a** Heatmap shows the H3K27me3 pattern around the transcription start site (TSS) region. Each panel represents 0.5 kb upstream and downstream of the TSS. **b** Heatmap of H3K4me3 and H3K27ac ChIP-seq signals in cells treated with DMSO and J4 at promoter-proximal regions. Rows are sorted by exclusive occupancy in the J4 condition. Cluster 1 in the metaplots refers to the occupancy particular in the DMSO condition, and cluster 2 represents the inverse comparison. **c** Venn diagram showing the overlap of upregulated genes by J4 among H3K4me3 and H3K27ac ChIP-seq peak. **d** GO analysis of 2092 commonly shared genes from H3K4me3 and H3K27ac ChIP-seq. **e** Genome browser tracks of H3K27me3, H3K4me3, and H3K27ac occupancy and RNA-seq at the *CHOP* gene locus. **f** RNA-seq analysis of differentially expressed genes in SJSA-1 cells treated with different drug formulations. **g** Venn diagrams showed the overlaps of differential genes of the three groups compared to the control. **h** Gene Set Enrichment Analysis (GSEA) showed that ER stress-associated genes were enriched in dual-drug-specific differential genes. **i** Fold change in ER stress-associated genes in SJSA-1 cells treated with different formulations for 48 h by qPCR analysis. **j** Western blot analysis of CHOP, GADD34, and ATF4 expression after the treatment with free dual drug or NP_J4+Apa_ (J4 5 μM; Apa 10 μM) for 48 h. β-Actin was used as the internal control. ***P < 0.001, **P < 0.01, or *P < 0.05

To gain a mechanistic insight into the effects of J4 on osteosarcoma cells, we performed chromatin immunoprecipitation sequencing (ChIP-seq) assay to investigate changes in H3K27me3, H3K4me3, and H3K27ac occupancy on associated genes in 143B cells treated with J4 for 48 h. As expected, we observed a global increase in H3K27me3 at the promoter regions upon J4 treatment (Fig. 3a). Importantly, we observed that J4 treatment also resulted in increased H3K4me3 and H3K27ac at specific gene loci (Fig. 3b). H3K4me3 and H3K27ac modifications at promoters and/or enhancers are important for gene activation. The Venn diagram (Fig. 3c) and gene ontology (GO) analysis (Fig. 3d) of the activated genes with H3K4me3 and H3K27ac modification revealed enrichment in the pathways involved in apoptosis in response to ER stress. *CHOP*, growth arrest and DNA damage-inducible protein 34 (*GADD34*), activating transcription factor 4 (*ATF4*), and *CEBPB* are the markers of ER stress.^19^ We found prominently increased H3K4me3 and H3K27ac ChIP-seq peaks and RNA-seq peaks at these ER stress marker gene loci upon J4 treatment (Fig. 3e, Supplementary Fig. 3a).

To further explore the mechanism underlying the synergistic efficacy of J4 and Apa against osteosarcoma, high-throughput RNA sequencing (RNA-Seq) of J4- and/or Apa-treated SJSA-1 cells was performed for comparison. There were far more differential gene expression changes in the J4 and Apa co-treated cells (>1723 genes altered; *P* < 0.05) than in the cells treated with J4 alone (<298 genes altered; *P* < 0.05) and Apa alone (<266 genes altered; *P* < 0.05) (Fig. 3f). Subsequent analyses of the overlapping differentially expressed genes indicated that many of these changes in gene expression in the dual-drug-treated cells were unique and specific (Fig. 3g). Gene Set Enrichment Analysis (GSEA) showed that the ER stress genes were enriched in the specific differentially expressed genes of the dual-drug-treated cells (Fig. 3h). Real-time PCR and immunoblot assays showed that the CHOP signaling genes were markedly increased in the dual-drug group compared with that in the control and single-drug groups (Fig. 3i, j, Supplementary Fig. 3b). In addition, dual-drug treatment also effectively induced the expression of the pro-apoptotic genes encoding BCL2 associated X, apoptosis regulator (*BAX*), Fas cell surface death receptor (*FAS*), and caspase 3 (*CASP3*), but decreased the anti-apoptotic gene encoding the BCL2 apoptosis regulator (*BCL2*) (Supplementary Fig. 3c). The expression of cell cycle-related genes encoding cyclin-dependent kinase 4 (*CDK4*) and cyclin-dependent kinase inhibitor 1A (*CDKN1A*) was also significantly altered in the dual-drug group (Supplementary Fig. 3d), which was consistent with previous results showing that this drug combination induced cell cycle blockade in the S phase.

### Osteosarcoma stem/progenitor cells display robust resistance to ER stress, which supports their survival in combined therapy

**Fig. 4:**
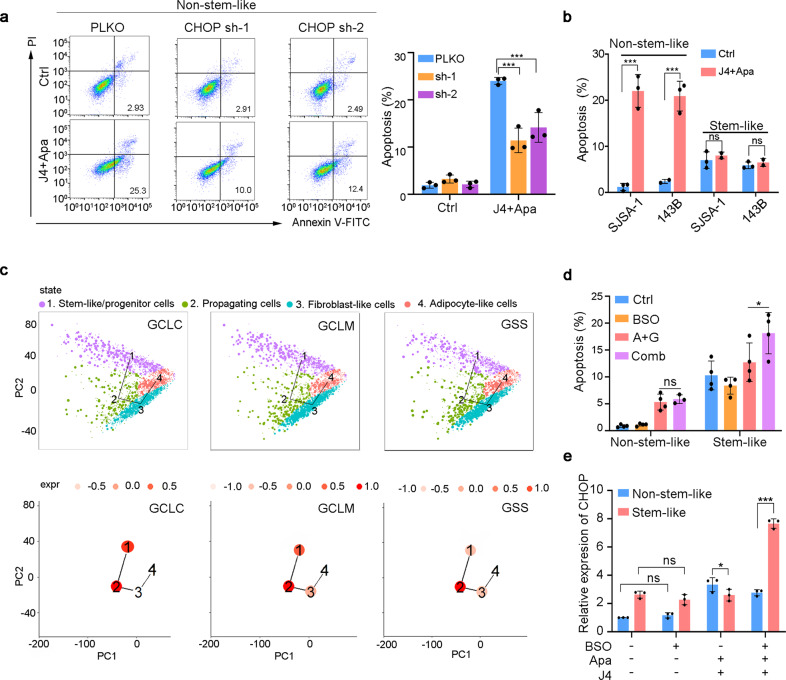
Osteosarcoma stem-like/progenitor cells demonstrate a poor response to ER stress. **a** Annexin V/PI staining of J4 and Apa-treated non-stem-like SJSA-1 cells or control cells transduced with or without CHOP shRNA. **b** Percentage of apoptotic cells in non-stem-like or stem-like cultured SJSA-1 or 143B treated with 5 μM J4 and 10 μM Apa at 48 h. **c** (up) Pseudo-time ordering of distinct populations within osteosarcoma cells. The clusters of cells are indicated with different colors. (down) The mean expression of GSH synthesis-related genes in each tree node. Expression levels (expr) are color coded. **d** Apoptotic cells were counted in non-stem-like or stem-like 143B cells conducted with different groups at 24 h. Comb, combination of J4 and Apa. **e** mRNA expression of CHOP in non-stem-like or stem-like 143B cells treated with multiple formulations

We next assessed the effects of J4 and Apa combination therapy on apoptosis in osteosarcoma cells. As expected, J4 and Apa co-administration markedly enhanced apoptosis in both cell lines (Supplementary Fig. 4a). Importantly, *CHOP* inhibition using shRNA blocked the effects of J4 and Apa on apoptosis (Fig. 4a, Supplementary Fig. 4b). However, when we tested the effect of J4 and Apa on the ability of stem-like osteosarcoma cells to form colonies of spheres, we found that the drug combination was unable to decrease the cell viability or the sphere formation capacity in the cells (Supplementary Fig. 4c, d). Furthermore, the drug combination treatment did not induce apoptosis of the sphere-forming stem-like osteosarcoma cells (Fig. 4b).

Previous studies have indicated that the GSH antioxidant pathways drive tumor resistance to ER stress. The key enzyme in GSH synthesis is the rate-limiting enzyme glutamate cysteine ligase (GCL), which is composed of a catalytic (GCLC) subunit and a modifier (GCLM) subunit. *GCLC* and *GCLM* were commonly overexpressed in quiescent and propagating stem-like/progenitor subpopulations in therapy-resistant osteosarcoma (Fig. 4c). Further dissection of the second enzyme of GSH synthesis, GSH synthetase (GSS), indicated that *GSS* was elevated in propagating osteosarcoma stem-like/progenitor cells (Fig. 4c). Moreover, J4 and Apa combination treatment increased the expression of *GCLC* and *GCLM*, but not *GSS* in 143B cells (supplementary Fig. 4e). Treatment with l-buthionine-sulfoximine (BSO), a GSH synthesis inhibitor, significantly decreased the intracellular GSH levels in stem-like osteosarcoma cells (Supplementary Fig. 4f). Combined treatment with J4 and Apa significantly induced apoptosis in stem-like osteosarcoma cells, while BSO had no additive apoptosis inducing effect on non-stem-like osteosarcoma cells (Fig. 4d). Of note, although BSO alone exerted no effect, the co-treatment of BSO with J4 and Apa significantly promoted *CHOP* expression in stem-like osteosarcoma cells (Fig. 4e). These studies provided functional evidence that GSH constitutes antioxidant defenses to render stem-like osteosarcoma cells invulnerable to ER stress-induced apoptosis by the combined treatment.

### Synthesis and characterization of nano-drugs

To circumvent this problem, we developed Cys-PDSA polymer-based NPs as drug carriers to facilitate GSH clearance in tumor cells. The formula for NP synthesis is illustrated in Supplementary Fig. 5a. As previously reported, Cys-PDSA polymers were prepared through the one-step rapid polycondensation of two nontoxic building blocks: l-cysteine esters and versatile fatty diacids.^20^ These polymers are denoted as Cys-nE (*n* = 2, 4, 6, 8, 10), with *n* representing the number of methylene groups in the diacid repeat unit, and *E* indicating the methyl ester of carboxylic acid on the side chain. The synthetic product was characterized using ^1^H NMR (Supplementary Fig. 5b), which verified the chemical structure of Cys-8E. As shown in Supplementary Fig. 5b, the appearance of proton peaks indicated the successful conjugation of the amide bond onto the methoxypoly (ethylene glycol)-conjugated Cys-PDSA backbone. Fourier-transform infrared spectroscopy was used to further confirm the structure, whereupon the unimodal bond of R_2_NH at 3300 cm^–1^ was found; the signal peak at 500 cm^–1^ was regarded as the presence of a polysulfide bond (Supplementary Fig. 5c). Then, a simple and efficient nanoprecipitation method was used for constructing the drug delivery platform. The encapsulation efficiency (EE of J4 and Apa reached 98.2 ± 1.1% and 96.4 ± 1.6%, respectively. The drug loading capacity (LC%) was 8.5 ± 1.2% of J4 and 21.4 ± 3.3% of Apa, respectively, as measured using high-performance liquid chromatography (HPLC) (Table 1). Cys-8E NP efficiently reduced intracellular GSH in stem-like osteosarcoma cells (Supplementary Fig. 5d).

**Table 1:** Characterization of NP_J4+Apa_

Tested samples	Diameter (nm)^a^	Polydispersity index^a^	Zeta potential (mV)^a^	EE of J4^b^ (%)	LC of J4^b^ (%)	EE of Apa^b^ (%)	LC of Apa^b^ (%)
NP_J4+Apa_	139.3 ± 17.6	0.15 ± 0.08	−22.5 ± 0.9	98.2 ± 1.1	8.5 ± 1.2	96.4 ± 1.6	21.4 ± 3.3

^a^Determined via DLS ^b^Analyzed via HPLC

**Fig. 5:**
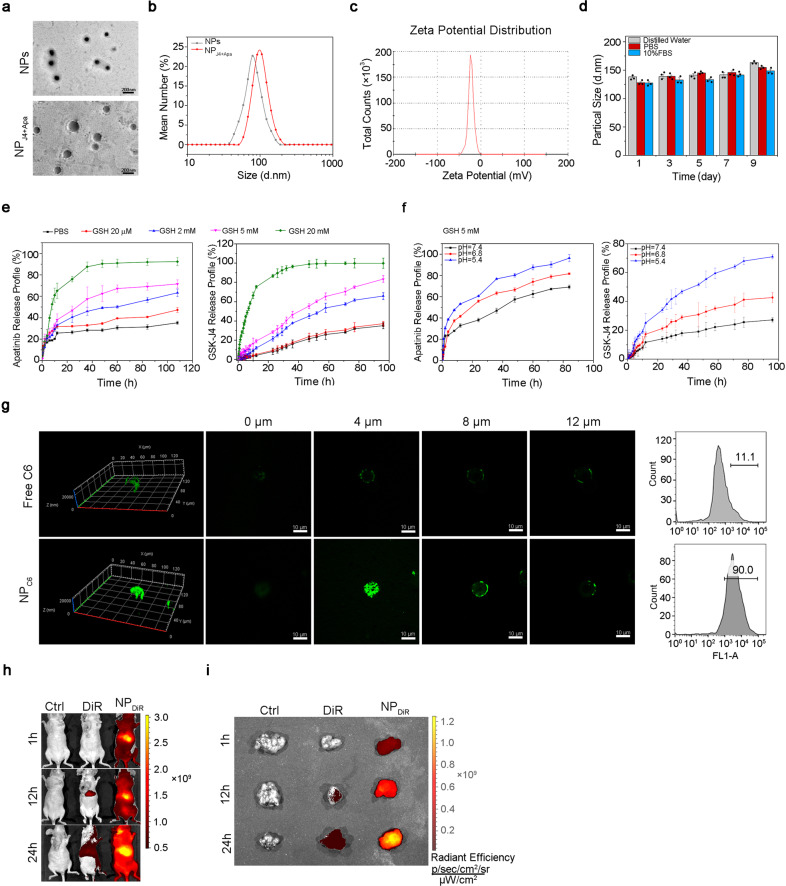
Characterization of NPs and NPJ4+Apa. **a** TEM of GSH-sensitive NPs (upper) and NP loading with J4 and Apa (NP_J4+Apa_, lower). The scale length of the TEM image is 200 nm. **b** DLS micrographs of NPs and NP_J4+Apa_. **c** Zeta potential of NP_J4+Apa_ dispersed in DI water. **d** Particle size of NP_J4+Apa_ in DI water, PBS, or 10% serum in DMEM solutions measured using DLS. **e** In vitro Apa (left) and J4 (right) release from NP_J4+Apa_ in PBS at different concentrations of GSH. **f** In vitro Apa (left) and J4 (right) release from NP_J4+Apa_ in PBS at different pH. **g** Penetration of coumarin-6-loaded NPs (NP_C6_) in the SJSA-1 and 143B-derived OS spheroids. The spheroids were incubated with free C6 or NP_C6_ for 30 min (green, C6). Flow cytometric quantification of C6 fluorescence intensity after treatment. The scale length of each image is 25 μm. **h** Representative in vivo fluorescence images of mice taken at three different time points (0, 12, and 24 h) after intravenous injection of saline (Ctrl), free DiR, or NP_DiR_. **i** The ex vivo fluorescence image of isolated tumors at 24 h post-injection

Transmission electron microscopy observations revealed that the NPs before and after J4 and Apa loading were spherical (Fig. 5a). Dynamic light scattering (DLS) results showed that the hydrodynamic radius (Rh) of blank NPs was 83.3 ± 18.9 nm (Fig. 5b). The higher Rh value for NP_J4+Apa_ than for the blank NPs indicated that the drugs had been successfully loaded onto the constructed platform. When the NP_J4+Apa_ particles were dispersed in deionized (DI) water, their zeta potential values were between –20 and –25 mV, whereas the blank NPs were electrically neutral (Fig. 5c). The appearance of these two formulations is shown in Supplementary Fig. 5e. DLS measurement of the stability of NP_J4+Apa_ in DI water, phosphate-buffered saline (PBS), or 10% serum-containing Dulbecco’s modified Eagle’s medium (DMEM) (Fig. 5d, Supplementary Fig. 5f) showed that the hydrodynamic diameters increased slightly after 9 days.

High intracellular concentrations of GSH (2–10 mM) facilitate the degradation of disulfide bonds.^21^ Extracellular concentrations of GSH are 100–1000 times lower (2–10 μM) than the intracellular concentrations.^22^ Under a high concentration of GSH, the disulfide bonds in the Cys-8E polymer would decompose, thus releasing the drug molecules. The results indicated that NP_J4+Apa_ could release approximately 95% of J4 and Apa in the presence of 20 mM GSH (Fig. 5e). Additionally, the NP_J4+Apa_ response to pH was evaluated at 5 mM GSH, mimicking the microenvironment within the tumor tissue. As shown in Fig. 5f, an obvious acceleration of J4 or Apa release was observed upon adjustment of the pH from 7.4 to 5.4.

### Cellular uptake of nano-drugs in vitro and tumor targeting efficiency in vivo

To investigate the role of the GSH-sensitive NPs in mediating the cellular uptake of drugs, Coumarin-6 (C6) was used as a model drug because its fluorescence enables easy quantitative and qualitative analyses. The cellular uptake of the C6-loaded NPs (NP_C6_) by the SJSA-1 and 143B cells was significantly higher than that of free C6 (Supplementary Fig. 5g). Figure 5g shows that NP_C6_ penetrated deeper into and was distributed more extensively in the SJSA-1- and 143B-derived spheroids than free C6. This suggested that the NPs had played a role in mediating the cellular uptake of C6.

To assess the biodistribution of the GSH-sensitive NPs, whole-animal imaging and ex vivo imaging of the major organs in the tumor-bearing mice were performed 24 h after the intraperitoneal injection of either free 1,1-dioctadecyl-3,3,3,3-tetramethylindotricarbocyanine iodide (DiR, a fluorescent lipophilic tracer) or DiR-loaded NPs (NP_DiR_). Noninvasive DiR fluorescence imaging showed a much more efficient accumulation of NP_DiR_ than free DiR at the tumor site at all time points from 0 to 24 h following administration (Fig. 5h). This result was confirmed by imaging the subcutaneous osteosarcoma tumors extracted from the mice at 24 h post-injection (Fig. 5i).

### Scavenging GSH renders resistant osteosarcoma vulnerable to synergistic treatment

**Fig. 6:**
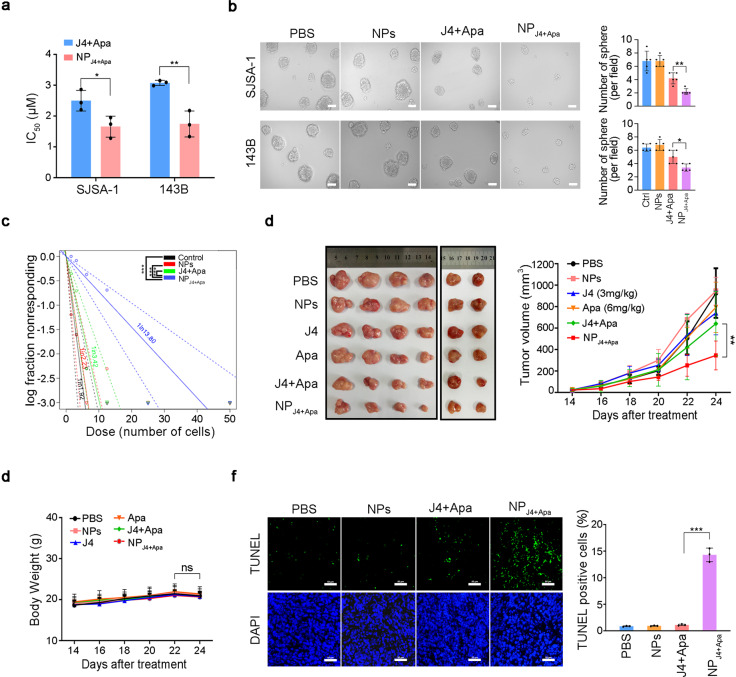
In vitro and in vivo antitumor efficacy of NPJ4+Apa by targeting osteosarcoma stem-like/progenitor cells. **a** IC_50_ of NP_J4+Apa_ and free drugs in SJSA-1 or 143B cells. **b** Morphology (left) and quantitative chart (right) of SJSA-1- or 143B-derived osteosarcoma spheroids treated with PBS, NPs, free drugs, and NP_J4+Apa_ on day 7, respectively, at J4 concentrations of 5 μM and Apa concentration of 10 μM. **c** The limiting dilution assay was used to evaluate the self-renewal capacity of SJSA-1 spheroid-derived osteosarcoma stem-like/progenitor cells treated with different formulations. **d** SJSA-1 spheroid-derived cells (stem-like) were subcutaneously injected into the right hind leg to establish osteosarcoma stem cell-derived xenografted tumors. (left) Representative photographs of the tumors when the nude mice were implanted with SJSA-1 spheroid-derived cells and further treated with different formula for six times. J4 (3 mg/kg), Apa (6 mg/kg), J4 (3 mg/kg) plus Apa (6 mg/kg) group, and NP_J4+Apa_ (J4, 3 mg/kg; Apa 6 mg/kg) groups. *N* = 6 in each group. (right) The average tumor volume in each group was measured at a different time point. **e** Cumulative body weight of mice during treatment. **f** TUNEL results of the tumor tissues from each treated mice group. Magnification ×200. *P* values were calculated using Student’s *t*-test (***P < 0.001, **P < 0.01, or *P < 0.05)

As we expected, NP_J4+Apa_ has GSH-scavenging effects on 143B-derived tumor spheroids (Supplementary Fig. 6a).^23^ We next studied the growth inhibitory effect of NP_J4+Apa_ on osteosarcoma stem-like/progenitor cell-derived spheroids and tumors. First, the effects of NP_J4+Apa_ on the viability of SJSA-1 and 143B cell monolayers were examined using the CellTiter-Glo assay. Treatment of the cells with NP_J4+Apa_ was highly effective in reducing their viability, with a 50% inhibitory concentration of 1.5 µM (IC_50_ = 1.5 µM), which was approximately 1.6-fold lower than the IC_50_ value of the free drugs (Fig. 6a). To evaluate whether the novel NP_J4+Apa_ specifically targets osteosarcoma stem-like/progenitor cells, sphere formation assays were performed. The spheroids exposed to the drug-free medium continued to grow throughout the experimental period and became tightly organized. In contrast, those incubated with NP_J4+Apa_ became distorted, with many cells dissociating from the spheres, and the growth of whole spheroids was significantly inhibited (Fig. 6b). In addition, NP_J4+Apa_ showed better performance of sphere formation inhibition compared with a non-GSH-responsive NP formulation with J4 and Apa (Supplementary Fig. 6b).

The most important properties of osteosarcoma stem-like/progenitor cells are their capacities for self-renewal and tumorigenesis.^24,25^ NP_J4+Apa_ significantly decreased the stem cell frequency in osteosarcoma stem-like/progenitor cell spheroids (Fig. 6c), whereas free drugs did not have any effect, even under the co-treatment. In addition, NP_J4+Apa_ significantly inhibited the expression of osteosarcoma stem cell signatures, such as *CD133*, *CD117*, and *SOX2*, in 143B-derived tumor spheroids (Supplementary Fig. 6c). The dramatic effect of NP_J4+Apa_ on osteosarcoma stem-like/progenitor cell self-renewal in vitro prompted us to test whether the effect could be replicated in vivo. To this end, SJSA-1 cell-derived spheroids were subcutaneously transplanted into nude mice. The NP_J4+Apa_-treated mice exhibited significantly higher antitumor activity than the mice treated with free dual drugs or empty NPs (Fig. 6d). Apparent adverse effects did not emerge, as the weights of the mice barely changed during the 24 days of observation (Fig. 6e). As shown by the terminal deoxynucleotidyl transferase-mediated dUTP-biotin nick end labeling (TUNEL) results in Fig. 6f, NP_J4+Apa_ treatment induced more apoptosis in the tumor tissue than the free drugs. Altogether, these tests provided evidence for the improved anti-osteosarcoma stem-like/progenitor cell efficacy of the J4 and Apa combination treatment, as facilitated by the GSH-sensitive NPs.

Some studies have revealed that NPs may exhibit a tendency to aggregate in the liver.^26,27^ In our study, in vivo imaging indicated a high uptake of NP_DiR_ in the liver, whereas other organs showed minimal uptake of the NPs. To evaluate the consequence of NP accumulation in the liver, H&E staining of the main tissues from SJSA-1 tumor-bearing mice (Supplementary Fig. 6d) was performed. The results showed that the main organs (heart, liver, spleen, lungs, and kidneys) remained the same in all groups. Moreover, hematological study of all the animals indicated that NP_J4+Apa_ did not increase the alanine aminotransferase (ALT) and aspartate transaminase (AST) levels, the upregulation of which is usually an indication of possible liver injury (Supplementary Fig. 6e). In summary, the GSH-scavenging NP_J4+Apa_ demonstrated excellent biocompatibility in vivo and remarkable antitumor efficacy.

## Discussion

The current treatment for primary osteosarcoma is a combination of surgery and chemotherapy.^28^ However, osteosarcoma “cancer stem-like cells” are thought to be resistant to conventional chemo-drugs, resulting in tumor recurrence. Our single-cell transcriptomics of therapy-resistant osteosarcoma identified a stem-like/progenitor bulk overexpressing JMJD3 and VEGFR2. The simultaneous inhibition of JMJD3 with J4 and of VEGFR2 with Apa had profound effects on key ER stress-related genes, leading to osteosarcoma cell apoptosis. Furthermore, we have shown that our GSH-scavenging NPs can efficiently accommodate J4 and Apa to significantly inhibit the survival of osteosarcoma stem-like/progenitor cells. To the best of our knowledge, this is the first study to characterize the cellular organization of chemotherapy-resistant cells in osteosarcoma tumors and eliminate osteosarcoma stem-like/progenitor cells using nano-drugs.

The existence of osteosarcoma cancer stem-like cells was first demonstrated by Gibbs et al.,^29^ who identified a subpopulation of osteosarcoma cells capable of growing osteospheres. Our study revealed that the actively propagating stem-like/progenitor cells can be derived from a quiescent stem-like cancer cell pool that acquires stemness-related properties via VEGFR2 signal stimuli. Moreover, epigenetic regulators, such as JMJD3 govern cancer stem-like functions. JMJD3 is upregulated in different human cancers, such as gliomas, breast cancer, and lung cancer.^30–33^ Thus, it is a novel target for the treatment of various types of cancer. Previous studies, including our work, showed that JMJD3 plays an important role in cell fate commitment, such as cellular reprograming into the pluripotent state.^34^ Further investigation is needed to determine whether inhibition of JMJD3 contributes to blocking the reprograming of endothelial-like cells into propagating cells.

The importance of a synergistic strategy is being increasingly realized for tumor treatment.^35,36^ Previous work has shown that J4 at 50–100 mg/kg was effective in inhibiting high-risk neuroblastomas in vivo. Herein, we have reported that a combination of J4 and Apa at 3–6 mg/kg could synergistically inhibit osteosarcoma tumor growth. Recent clinical trial data have shown that Apa has promising efficacy against various types of cancers, with acceptable toxicity. Compared with the findings in previous reports, the intake of J4 and Apa in our study is considered much safer for long-term treatment and has fewer side effects.^37^ Hence, the coupled J4 and Apa may represent a potent drug pair against osteosarcoma tumor in the clinic.

Our RNA-seq and ChIP-seq data suggested the importance of ER stress-induced apoptosis in targeting osteosarcoma cells. The relief of ER stress by CHOP knockdown in this study effectively inhibited the ability of the combination treatment to induce apoptosis, indicating that additional ER stress plays an important role in eliminating these tumor cells. Moreover, we found that osteosarcoma stem-like/progenitor cells have adapted to gain a survival advantage by enhanced GSH synthesis to maintain the resistance to ER stress-induced apoptosis. Therefore, osteosarcoma therapy could be targeted to tilt the balance of GSH/GSSG (oxidized glutathione) toward oxidation by driving malignant cells to a terminal ER stress response. It is worth noting that since apoptosis is an early response cell death, the late-response cell death or nonapoptotic cell death, autophagy, can also be observed after nanodrug treatment. Therefore, the overall tumor response in animal studies far exceeds the results of apoptosis in vitro.

Recently, several GSH-scavenging materials have been shown to be excellent and suitable platforms for triggering the clearance of GSH, thus potentially offering a more efficient potency of drugs.^38^ The major advantages of our GSH-scavenging NPs over traditional ones include greater drug-loading efficiency, faster drug release, and higher biosafety. In contrast to the existing GSH-sensitive NPs based on liposomes or hydrophilic nanocores, ours have hydrophobic nanostructures and a PDSA core, allowing the NPs to carry more drugs, especially J4 and Apa.^39^ With this method, it is now possible to reach the effective drug concentration of J4 and Apa in osteosarcoma stem-like/progenitor cells, so that the ER stress can exceed the threshold to induce cell apoptosis. In addition, the fixed drug proportion and fast intracellular release that our GSH-scavenging NPs provide, enabled J4 and Apa to induce cell apoptosis in a synergistic manner. Furthermore, we improved the safety of the NPs through their surface modification with disulfide groups to facilitate the GSH-scavenging process. Indeed, NPs loaded with or without the dual drugs exhibited no toxicity toward normal cells, based on the results of the histological and hematological studies of the major organs. However, regarding the clinical application, stricter biosafety assays for the approval of NP_J4+Apa_ might be required.

In summary, our GSH-scavenging nanoplatform can effectively deliver J4 and Apa into osteosarcoma stem-like/progenitor cells effectively, with implications for increasing the drug uptake in the tumor cells, optimizing the drug intracellular release time, and improving the synergistic effects on therapy-resistant osteosarcoma cells. Further clinical tests of the J4 and Apa combination will be required to systematically investigate their influence on therapy-resistant osteosarcoma to improve patient outcomes.

## Materials and methods

### Materials and reagents

All chemicals and solvents used were of analytical grade. J4 was obtained from Selleck Chemicals (USA). Apa was provided by Jiangsu Hengrui Medicine Co., Ltd (China). CellTiter-Glo® Luminescent Cell Viability Assay was purchased from Promega Biotech Co., Ltd. Annexin V-FITC/PI apoptosis kit was purchased from Lianke Technology Co., Ltd. Hematoxylin and eosin were purchased from Guangzhou Ying Ze Biotechnology Co., Ltd (China). Reduced GSH assay kit was obtained from Nanjing Jiancheng Bioengineering Institute. Matrigel® basement membrane matrix was purchased from Corning (USA). An enhanced BCA protein assay kit was purchased from Beyotime (China). TUNEL assay kit was obtained from Promega (Beijing) Biotech Co., Ltd, and 4% paraformaldehyde was purchased from Guangzhou Ruishu Biotechnology Co., Ltd. DAPI was acquired from Sigma-Aldrich. Lipofectamine 3000 reagent, TRIzol Reagent, and Power SYBR® PCR Master Mix were purchased from Life Technologies.

### Cells

SJSA-1 cells and 143B cells were cultured in DMEM (Corning, USA) containing 10% FBS and 1 mM l-glutamine. All cells were cultured at 37 °C in a humidified atmosphere of 5% CO_2_.

### Animals

Female Babl/c nude mice (20 ± 2 g, 5–6 weeks) were purchased from the Model Animal Research Center of Nanjing University (China). This study was carried out in accordance with the guidelines to avoid or reduce the suffering of laboratory animals. The animal research experiments were approved by the Ethical Committee of Zhongshan School of Medicine.

### Synthesis of NP_J4+Apa_

To prepare Cys-8E NPs and drugs, Cys-8E were dissolved in dimethyl sulfoxide (DMSO) at a concentration of 20 mg/mL to form oil phase 1, as well as drugs Apa and J4 were dissolved in DMSO at a concentration of 9 mg/mL, and the quality ratio of Apa to J4 was 2:1. Stabilizer DSPE-PEG 2000 or 3000 was dissolved in DMSO at a concentration of 4 mg/mL to form oil phase 2. Two oil phases were mixed to keep the stabilizer 20% (weight) of Cys-8E and drugs and shake for seconds, then dropped into stirring distilled water at a speed of 2000 r.p.m. The ratio of the oil phase to the water phase is 1:9. In order to remove the remaining free molecules and organic DMSO solution, centrifugal operation (2500 r.p.m., 10 min) was carried out three times using a centrifugal filter with a molecular weight cutoff of 100,000 Da. Finally, a concentrated PBS buffer was added to obtain a final 1× concentration of PBS.

### Characterization of NPs and NP_J4+Apa_

The NP size, polydispersity index, and zeta potential were measured by DLS. The morphology of the NPs was characterized by transition electron microscopy (TEM, JEM-1400 Plus, 120 kV, JEOL). The process of NP_coumarin 6_ and NP_DiR_ preparation was the same as that of NP_J4+Apa_. The concentration of dye contained in the final NPs was 20 μg/mL. To evaluate the stability of the NPs, the NPs were dispersed in distilled water, PBS, and 10% FBS DMEM media. The changes in particle size in each group of NPs were measured using DLS every 2 days. The drug LC and EE were measured with HPLC (Agilent 1260 Infinity II, USA). Specifically, an aliquot of NP samples was dissolved in tetrahydrofuran and centrifuged at 13,000 r.p.m. for 10 min. The supernatants were then injected into the HPLC column for the drug content analysis. The DL and EE were calculated as follows:

$${\mathrm{LC}}\,(\% ) = \frac{{{\mathrm{the}}\,{\mathrm{weight}}\,{\mathrm{of}}\,{\mathrm{encapsulated}}\,{\mathrm{J4 + Apa}}}}{{{\mathrm{the}}\,{\mathrm{total}}\,{\mathrm{weight}}\,{\mathrm{of}}\,{\mathrm{nanoparticles}}}} \times 100{\mathrm{\% }},$$

$${\mathrm{EE}}\,(\% ) = \frac{{{\mathrm{the}}\,{\mathrm{weight}}\,{\mathrm{of}}\,{\mathrm{encapsulated}}\,{\mathrm{J4 + Apa}}}}{{{\mathrm{the}}\,{\mathrm{total}}\,{\mathrm{weight}}\,{\mathrm{of}}\,{\mathrm{drug}}}} \times 100{\mathrm{\% }}.$$

### Drug-releasing behavior of NP_J4+Apa_

The drug release of NP_J4+Apa_ was tested under different GSH concentrations or different pH values. First, 1 mL of concentrated NP_J4+Apa_ was placed in a dialysis bag (molecular weight cutoff (MWCO) = 3500). It was then put into different release media (pH 7.4) containing various GSH concentrations (0, 20 μM, 2 mM, 5 mM, 20 mM), and released in a 37 °C shaking table. At certain time intervals, 1 mL of PBS medium was aspirated, and another 1 mL of fresh PBS was added. The amount of Apa and J4 NPs released outside the dialysis bag was determined using HPLC. On this basis, to make sure that the drug-releasing behavior under different pH values conditions was the same, the dialysis bags were put into PBS solutions with different pH values (pH 5.4, 6.8, and 7.4) when the GSH concentration is 5 mM, mimicking the microenvironment within the tumor tissue. Drug release at different pH levels was determined using HPLC.

### Single-cell RNA-seq data analysis

For our 10× Genomics Chromium scRNA-seq of osteosarcoma cells, we aimed to capture approximately 7000 cells. Raw data were processed using Cell Ranger (v3.0.2) to align reads, generate feature-barcode matrices, and perform gene expression analysis. We used the mkfastq pipelines to make Fastq files. We used the cell count pipelines for alignment (with reference genome Hg19), filtering, barcode counting, and UMI counting. The cells were normalized to the total UMI read count as instructed in the manufacturer’s manual (http://satijalab.org/seurat/). scRNA-seq data that met quality control criteria were used for transcriptomic analysis. t-SNE was performed for unsupervised clustering. The SingleR package was used for cell-type identification analysis. We used cell-type markers for the identification of specific cell types in t-SNE clusters. To analyze the trajectory development of tumor cells, the unsupervised pseudodevelopmental timeline of single cells was calculated using the package TSCAN^40^ and Monocle.^41^

### Cytotoxicity assay

Cell viability was evaluated using the CellTiter-Glo Luminescent Cell Viability Assay according to the manufacturer’s instructions. Briefly, cells were seeded in 96-well plates (Fisher Scientific) at a density of 3000 cells/well. After 24 h, cells were treated with drug-loaded NPs or free drugs dissolved in the medium. When measuring cell viability, the incubation medium was removed, and 50 μL of the CellTiter-Glo reagent was added into each well for 20 min incubation. The absorbance was read with a Synergy™ HTX multi-mode reader (BioTek). Half-maximum inhibitory concentrations (IC_50_ values) were calculated using GraphPad Prism 7.0.

### Flow cytometry

According to the manufacturer’s instructions, the cells were prepared, fixed, and incubated with Annexin V-FITC or PI for apoptosis and cell cycle analysis, respectively. Flow cytometry was performed using BD LSRFortessa (BD Biosciences, CA, USA). Analyses were performed using the FlowJo software.

### Preparation of tumor spheroids

To prepare tumor spheroids, SJSA-1 and 143B cells (5 × 10^3^ cells/well) were cultured in a Corning® 96-Well Ultra Low Attachment Microplate. After 5–7 days, uniform and compacted spheroids were selected for the following studies. The spheroid-derived cells were used as stem-like osteosarcoma cells. The monolayer cultured cells were used as non-stem-like osteosarcoma cells. For the uptake study, the tumor spheroids were incubated with free coumarin-6 or NP_coumarin 6_ for 30 min, and then the spheroids were rinsed with cold PBS and fixed with 4% paraformaldehyde for 15 min. Fluorescence intensity was observed under a fluorescent microscope and further quantified with flow cytometry (BD LSRFortessa). For the tumor spheroid formation assay, DMSO, J4, Apa, and J4+Apa were added to each well. The morphology of the spheroids was observed under a microscope. Cell apoptosis was determined using flow cytometry.

### Cellular uptake

NP_coumarin 6_ was obtained with 1.3 µg/mL coumarin-6 as described above. SJSA-1 cells and 143B cells were seeded in 96-Well Ultra Low Attachment Microplate, and then treated with different coumarin-6 formulations. After an incubation for 30 min, the cells were washed, fixed in 4% paraformaldehyde, and stained with DAPI. Images were obtained using a fluorescent microscope. Fluorescence intensity was further determined using flow cytometry (BD LSRFortesa).

### Tumor xenograft mouse studies

Five- to six-week-old Balb/c female nude mice were subcutaneously administered approximately 1 × 10^7^ SJSA-1 cells (overexpressing luciferase) into the right hind leg to establish xenografted tumors. To test the anti-osteosarcoma stem cell activity of NP_J4+Apa_, 1 × 10^7^ SJSA-1 spheroid-derived cells were subcutaneously injected into the right hind leg to establish osteosarcoma stem cell-derived xenografted tumors. Different formulations were intravenously administrated once every other day. The animal weights and tumor volumes were measured every day. Tumor size was calculated as the tumor volume = (tumor length) × (tumor width)^2^/2. For in vivo fluorescence imaging, the mice were injected with d-luciferin and anesthetized. The fluorescence signals were measured using the Xenogen IVIS Spectrum (Caliper Life Sciences, USA). After the mice were sacrificed, the tumor tissues were fixed and stained with hematoxylin and eosin (H&E) for histopathological evaluation.

For in vivo fluorescence imaging of DiR, 100 μL saline, 10 μg near infrared dye (DiR) in 100 μL saline, or 10 μg DiR-loaded NPs (NP_DiR_) in 100 μL saline was intravenously injected into tumor-bearing nude mice. The mice were anesthetized, and fluorescence signals were measured using the Xenogen IVIS Spectrum at 12 and 24 h post-injection. Three mice were used in each group. Then, fluorescence images of the whole bodies and tumors dissected from nude mice were individually taken as above.

### Limiting dilution assay

SJSA-1 cells were plated in 96-well plates at 1, 5, 10, 20, 50 cells/well/100 μL in DMEM/F12 medium with 1% B27, 10 ng/mL hEGF, and 10 ng/mL bFGF. The percentage of wells with spheres was determined after 7 days. Positive wells with sphere formation were counted and log fractions of wells without spheres were plotted, and data were calculated using the Extreme Limiting Dilution Analysis (http://bioinf.wehi.edu.au/software/elda/index.html).

### TUNEL assay

The TUNEL method was used to label the 3′-end of fragmented DNA of the apoptotic tumor tissue dissected from nude mice. The tumor tissues were stripped off and embedded in paraffin. After being cut into 5.0-mm sections, tumors were stained via TUNEL method, using an in situ cell death detection kit, POD (Roche), according to the manufacturer’s instructions. The number of TUNEL-positive cells in the tumor tissues was evaluated using images acquired in a fluorescent microscope.

### Total RNA isolation

After the treatment with different formulations, RNA was extracted using SV Total RNA Isolation System (Promega (Beijing) Biotech Co., Ltd) as per the manufacturer’s protocol. Samples were mixed with RNA lysis buffer, and RNA dilution buffer was added. After the centrifugation of the mixture for 10 min, 95% ethanol was used to clear the lysate and mix it well. The RNA wash solution was used before and after DNase incubation mix to obtain pure RNA. Total RNA was dissolved in nuclease-free water. RNA samples were quantified using a UV-VIS spectrophotometer NanoDrop 2000 (Thermo Fisher Scientific).

### GO analysis

GO analysis was performed based on the Database of Annotation, Visualization and Integrated Discovery (DAVID, https://david.ncifcrf.gov/). GO terms with Benjamini–Hochberg adjusted *P* value <0.05 were significantly enriched by J4+Apa. GO analysis was utilized to explore the potential functions and crucial pathways involved in J4 or/and Apa treatment.

### Gene set enrichment analysis

To confirm the transcriptomic signatures, the GSEA tool (v3.0) from the Broad Institute was used. GSEA was used to determine whether a range of previously defined gene sets were enriched in different phenotypes. Hallmark gene sets were acquired from the Molecular Signatures Database (MSigDB). Benjamini–Hochberg adjusted for *P* < 0.05 and false discovery rate <0.25 were used as significance cutoff criteria. The enrichment score indicated the degree to which a gene set was overrepresented at the top or bottom of a ranked list of genes.

### Statistical analysis

Data are shown as mean ± standard deviation of the mean as indicated. Statistical analysis was performed using GraphPad Prism 6.0. The difference between groups was analyzed using one-way analysis of variance. *P* < 0.05 was considered statistically significant.

## Supplementary information

Supplementary Information

## Data Availability

All data needed to evaluate the conclusions in the paper are present in the paper and/or the Supplementary Materials. Additional data available from authors upon request.
