# Supplementary material for: Nanoparticle enhanced combination therapy for stem-like progenitors defined by single-cell transcriptomics in chemotherapy-resistant osteosarcoma
Source: Signal Transduct Target Ther. 2020 Sep 25;5:196. doi: 10.1038/s41392-020-00248-x (PMC7518281; doi:10.1038/s41392-020-00248-x)
Supplement: Supplementary file 1 — Supplementary Information [file 41392_2020_248_MOESM1_ESM.docx]

Supplementary Materials for

Nanoparticle Enhanced Combination Therapy for Stem-like Progenitors Defined by Single-Cell Transcriptomics in Chemotherapy-Resistant Osteosarcoma

Li Wang, Xiaojia Huang, Xinru You, Tianqi Yi, Bing Lu, Jiali Liu, Guohao Lu, Minglin Ma, Changye Zou, Jun Wu, and Wei Zhao

Correspondence to: zhaowei23@mail.sysu.edu.cn (W. Zhao),

wujun29@mail.sysu.edu.cn (J. Wu),

**This PDF file includes:**

Materials and Methods

Figure. S1 to S6

Tables S1

**Materials and Methods**

Western blotting

The treated cells were harvested and lysed with the lysis buffer, supplemented with a protease inhibitor cocktail (Roche) and a phosphatase inhibitor (Roche) on ice. SDS sample buffer was added to achieve a 4-time dilution, and boiled for 5 min at 100℃. Proteins were separated by electrophoresis on an SDS-polyacrylamide gel and transferred to PVDF membrane (Millipore). After blocking with 5% nonfat dry milk for 1 h, the PVDF membrane was incubated overnight at 4 °C with a primary antibody, extensively washed, incubated with horseradish peroxidase-conjugated secondary antibody for 1 h, and finally visualized with an enhanced chemiluminescence (ECL) kit.

RT-qPCR assay

For the quantitative real-time PCR studies, a RNeasy Mini Kit (Qiagen) was used to isolate total RNA. Reverse transcription was performed using SuperScript III reverse transcriptase (Invitrogen). Quantitative real-time polymerase chain reaction (RT-qPCR) was performed using Power SYBR® PCR Master Mix (Life Technologies) in accordance with manufacturer's instruction. All reactions were triplicated. qPCR primers are detailed in Supplemental Table 1.

Pathological section analysis

Main organs in each group were fixed with 4% polyformaldehyde for 48 h, dehydrated and embedded in [paraffin](https://www.sciencedirect.com/topics/pharmacology-toxicology-and-pharmaceutical-science/paraffin) at 70℃. Paraffin sections were sliced in 5 μm sections, stained with H&E, observed and photographed under microscope. The histopathological changes of main organs in control group and drug treated groups were observed to estimate the integrity of tissue structure, degree of [degeneration](https://www.sciencedirect.com/topics/pharmacology-toxicology-and-pharmaceutical-science/degeneration), [necrosis](https://www.sciencedirect.com/topics/pharmacology-toxicology-and-pharmaceutical-science/necrosis) and inflammatory cell [infiltration](https://www.sciencedirect.com/topics/earth-and-planetary-sciences/infiltration).

Wound healing assay

The wound healing assay was applied to detect the effects of different formulations on the migratory activity of SJSA-1 and 143B cells. Cells were seeded at a density of 5 × 10^5^ cells per well in a 6-well plate and incubated until completely confluent. Then, a straight line was scratched with a 200 μL sterile pipette tip to make a wound. After rinsed with PBS, cells were further incubated with 0.5% fetal bovine serum /DMEM culture medium (controlled group), GSK-J4 (5μM), Apatinib (10μM) and J4+Apa (5μM, calculated using J4 concentration). At 0 h and 96 h, the width of the open wound was photographed by an optical microscope (Olympus, Tokyo, Japan). Cell migration ability was quantified by the relative wound width of cells treated with different formulations at 96 h was compared to that at 0 h (n = 3).

*In vitro* invasion assay

A cell invasion assay was conducted using a transwell chamber (Falcon® 24 Well Permeable Support Companion Plate; Corning). SJSA-1 and 143B cells (4 × 10^4^ per well) were seeded in the upper chamber, coated with growth factor reduced Matrigel (Corning), in 300 μl serum-free medium. The lower chamber was filled with 800 μl medium containing 10% FBS to induce cell migration. After incubated with different formulations for 24 h, cells in the upper surface of the membrane were removed with a cotton swab. Migrated cells, to the lower surface of the membrane, were fixed with methanol and stained with 0.5% crystal violet for 30 min. The images were obtained using a light microscope (Olympus) in five different view fields. The experiment was conducted in triplicate.

Serum liver function assay

Blood samples were obtained from nude mice after treated with PBS, NPs, J4+Apa (3mg/kg, calculated using J4 concentration) and equivalent NP_J4+Apa_ for 6 times. Serum alanine aminotransferase (ALT) and aspartate transaminase (AST) levels were measured according to a kit manual (Jiancheng Bioengineering Ltd, Nanjing, China).

Immunostaining of osteosarcoma specimens

The immunofluorescence staining was performed on 5 mm formalin-fixed paraffin embedded section of the chemotherapy-resistant osteosarcoma. Re-hydration and heat-mediated antigen retrieval was conducted in 10 mM sodium citrate buffer solution (pH 6). After blocking with 5% BSA for 30 min, the individual section was incubated with primary antibody against JMJD3 (abcam, cat#ab169197), c-MYC (affinity, cat#AF0358), or VEGFR2 (huabio, cat#RT1650), respectively, for overnight at 4℃. Antigen detection was conducted using the Alexa Fluor 488 (Thermo Fisher Scientific, cat#A32723) or Alexa Fluor 594 (Thermo Fisher Scientific, cat#A32740) secondary antibodies for 2 h at room temperature in the dark. After three washes in PBS, sections were incubated with Hochest33342 (Thermo Fisher Scientific). Images were obtained with a Zeiss LSM710 Multi-Photon Confocal Microscope and analyzed using ZEN software.


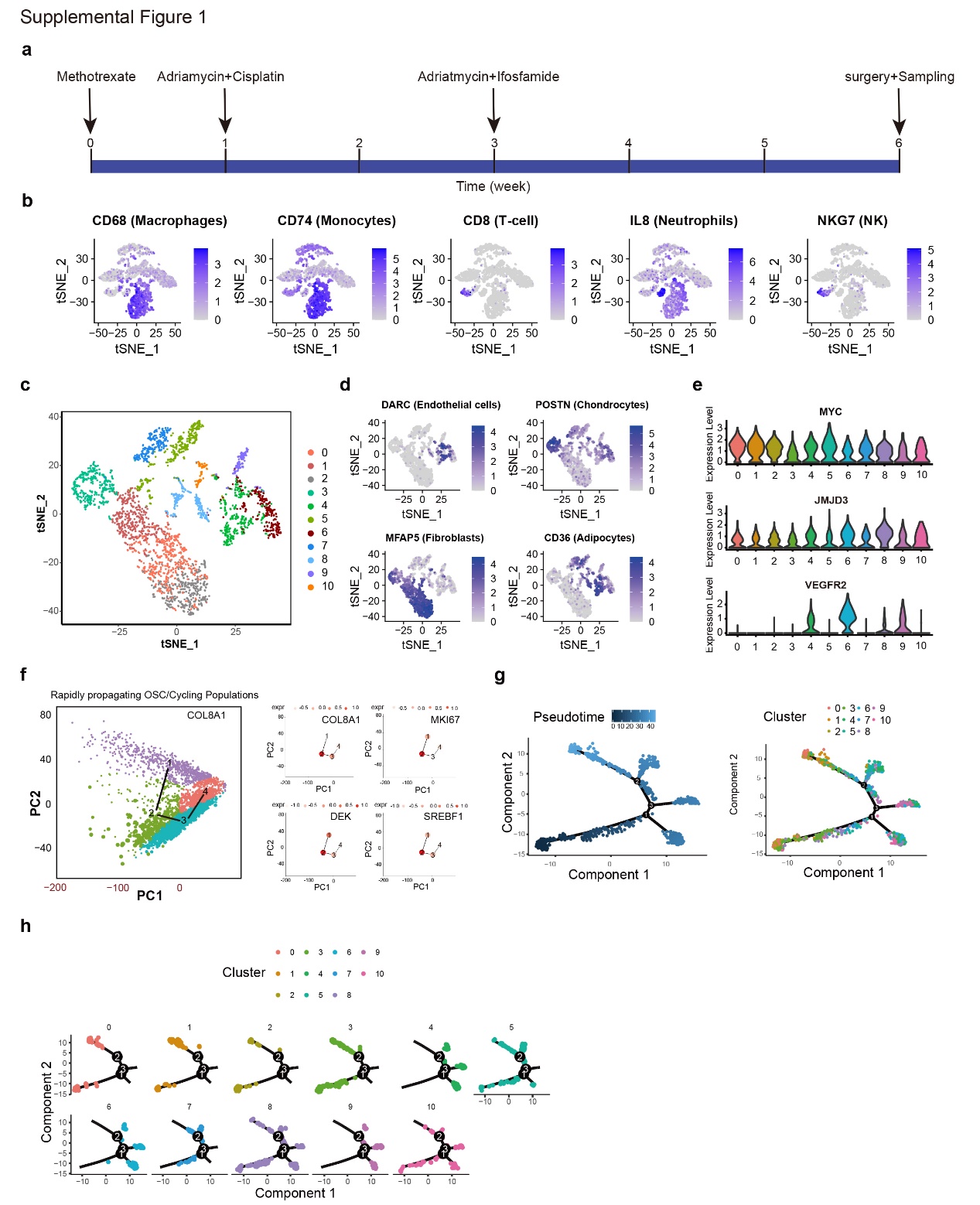


**Figure. S1.**

**Single-cell transcriptomics reveal heterogeneous cell populations and development hierarchy in osteosarcoma.** **Related to Figure 1. a** Timeline diagram of preparation for single cell sample. **b** t-SNE visualization of the expression of curated feature genes for immune cell clusters. **c** Re-clustering of the Osteosarcoma cells after Removing immune cells. (n=3,168). **d** Representative markers of each cluster within Osteosarcoma. **e** Violin plots revealed the expression pattern of *MYC*, *JMJD3,* and *VEGFR2* in endothelial-like populations (subpopulation 4, 6, and 9). **f** TSCAN plots showing the rapidly-propagating populations. The mean expression of three representative cycling genes (*SRSBF1*, G1 phase; *DEK*, S phase; *MKI67*, G2M phase), indicating that chondrocyte-like cells propagate after chemotherapy. **g-h** Pseudotime trajectory (via Monocle) of the osteosarcoma cells predicts subpopulation 4, 6, and 9 as early progenitors.


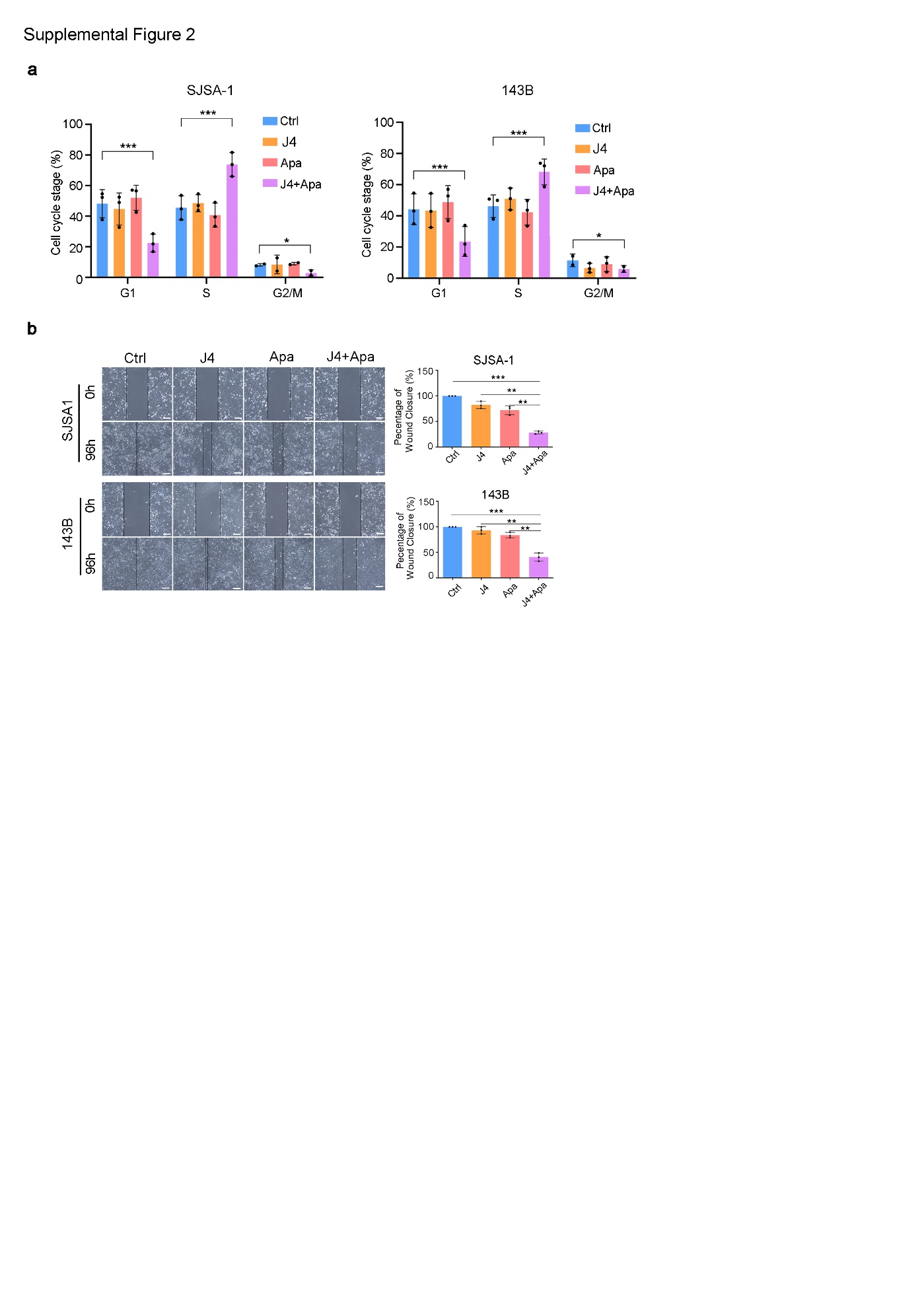
Figure. S2.

**Combined GSK-J4 (J4) and Apatinib (Apa) displayed prominent antitumor therapy. Related to Figure 2. a** Cell cycle analysis on SJSA-1 and 143B cells (non-stem-like) after treatment with J4 or/and Apa for 48 h. **b** Cell migration was measured by a scratch assay and monitoring the migration of SJSA-1 and 143B cells (non-stem-like) into the scratched area. Data are presented as percentage of cell migration, which indicates the scratched area at a given time as a proportion of the initial scratched area. Scale bar = 200 μm.

The data are expressed as mean ± SD. All results are from three independent experiments. P values were calculated by Student’s t test (***P < 0.001, **P < 0.01, or *P < 0.05).


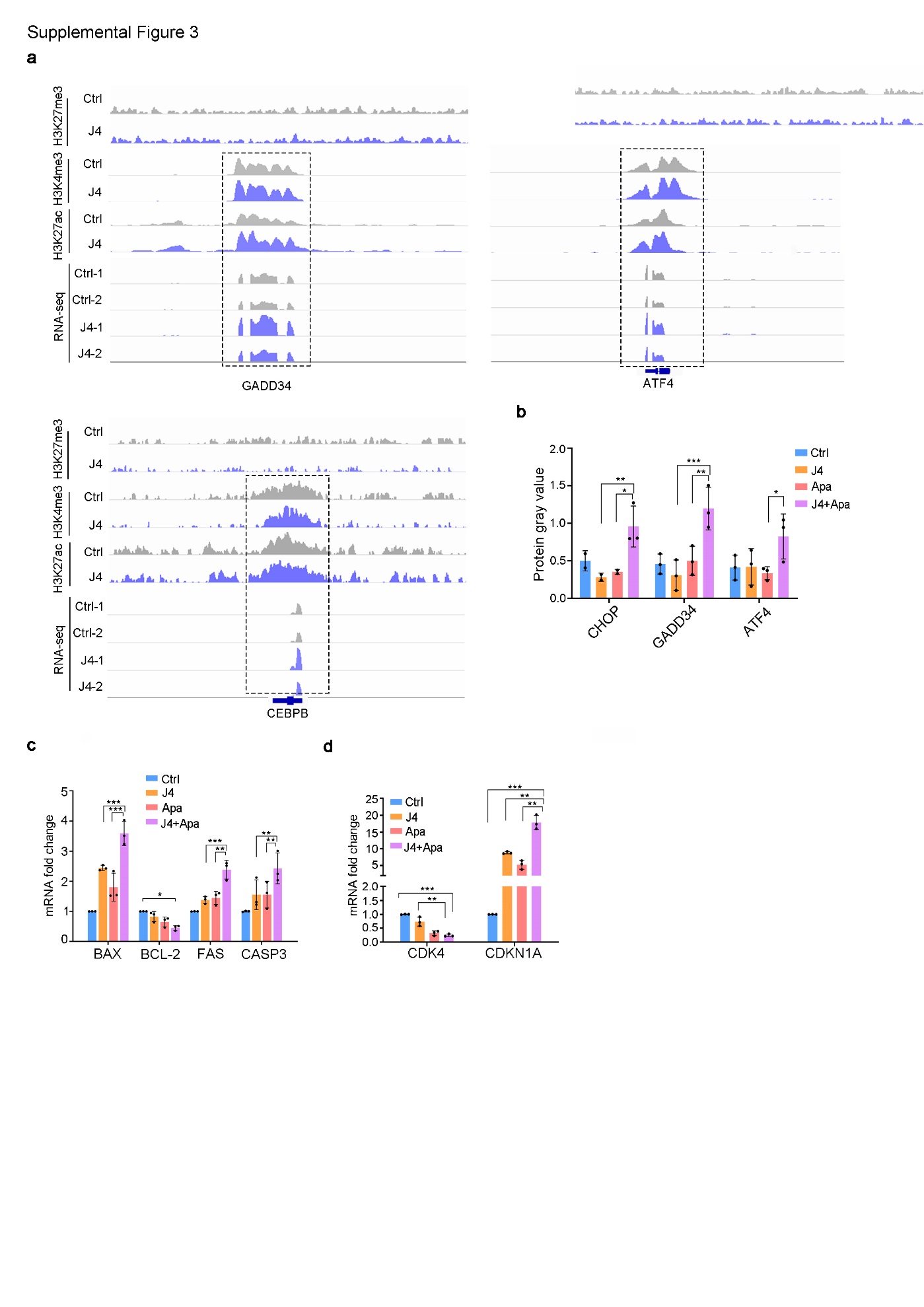
Figure. S3.

**Cooperative mechanism of the combination drugs. Related to Figure 3.** **a** Genome browser tracks of H3K27me3, H3K4me3 and H3K27ac ChIP-seq and RNA-seq coverage at the *GADD34*, *ATF4*, and *CEBPB* genes in the 143B cells after treatment with J4. Dotted line indicates the position of the annotated transcription end site (TES) site. **b** Protein gray value of western blot concerned GADD34, CHOP, and ATF4 after treatment with various formulations. **c** Fold change of apoptotic genes in SJSA-1 cell lines treated with different formulations for 48 hours by qPCR analysis. **d** qPCR analysis of cell cycle associated genes, *CDK4* and *CDKN1A*, identified from the RNA-seq data in SJSA-1 cell lines treated with different formulations for 48 hours. **b-d** Data are expressed as mean ± SD, and the results are from three independent experiments. P values were calculated by Student’s t test (***P < 0.001, **P < 0.01, or *P < 0.05).


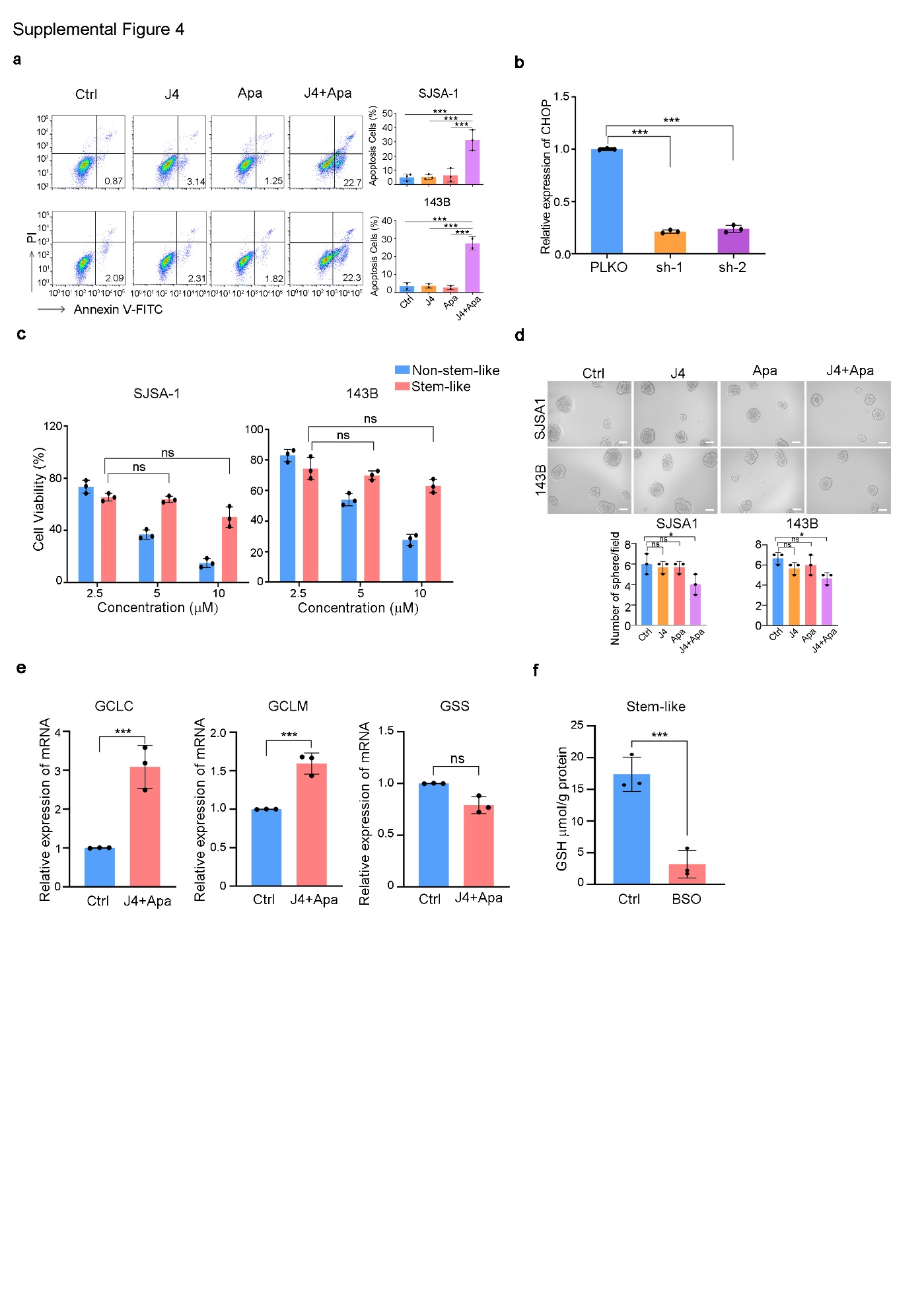


**Figure. S4.**

**Combination therapy exhibits noneffective in stem-like osteosarcoma cells. Related to Figure 4. a** Annexin V-FITC/PI staining for apoptosis in SJSA-1 and 143B cells treated with J4 or/and Apa for 48 h was assessed by flow cytometry analysis. Columns represent the average proportions of apoptotic cells. **b** mRNA expression of CHOP in CHOP shRNA transduced SJSA-1 cells. **c** Cell viability of non-stem-like and stem-like cells treated with J4 or/and Apa for 48h. **d** Morphology (left) and quantitative chart (right) of SJSA-1 or 143B derived OS spheroids treated with PBS, J4, Apa and J4 plus Apa respectively, at the J4 concentrations 5 μg/ml and Apa concentration 10 μg/ml. **e** The mRNA expression of *GCLC*, *GCLM,* and *GSS* in 143B cells treated with J4+Apa for 48 h. **f** Intracellular GSH concentration in stem-like 143B cells treated with BSO.

The data are expressed as mean ± SD. All results are from three independent experiments. P values were calculated by Student’s t test (***P < 0.001, **P < 0.01, or *P < 0.05).


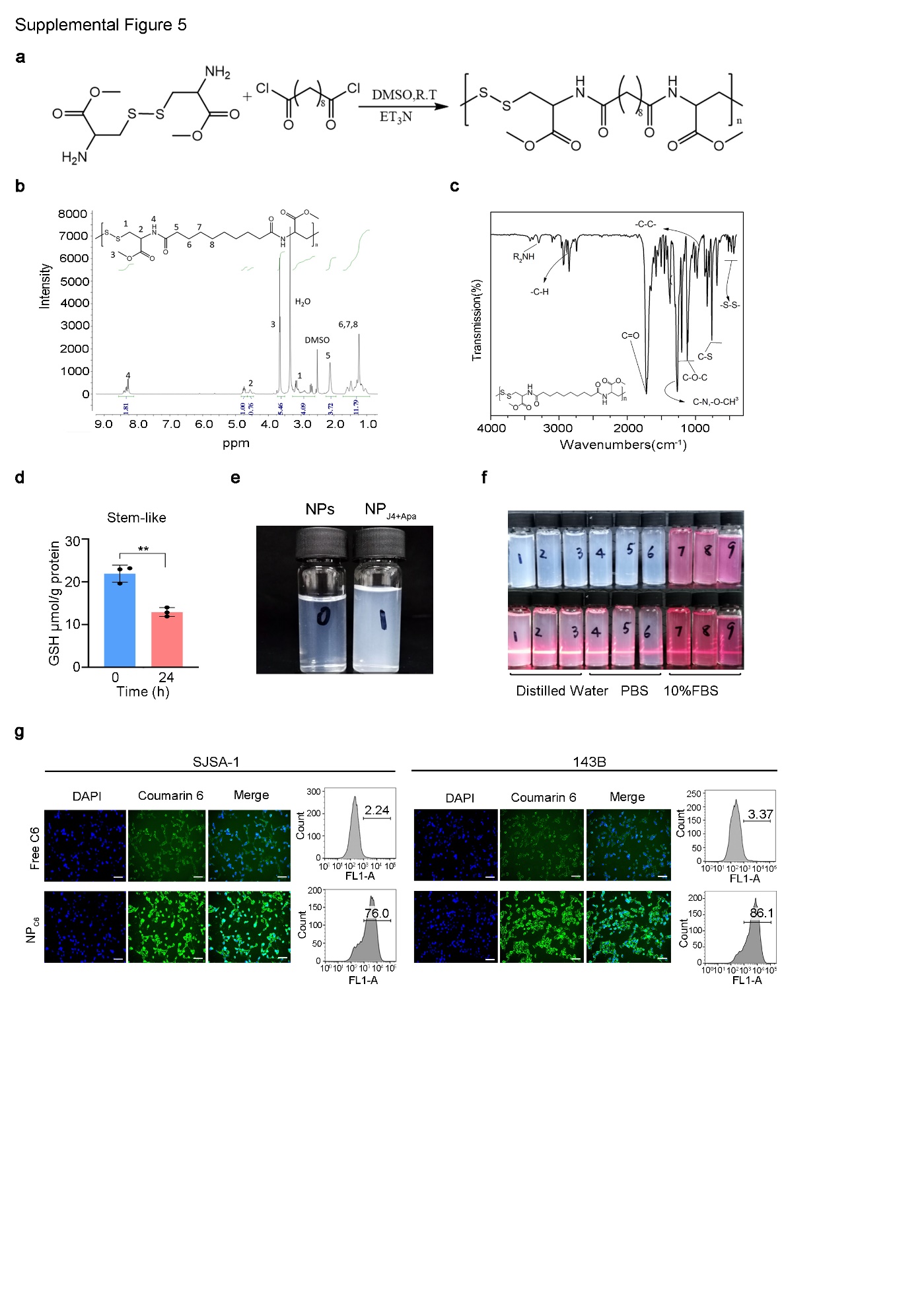


**Figure. S5.**

**Synthesis and characterization of Cys-8E polymer, NPs, NP_J4+Apa_. Related to Figure 5.** **a** Composition formula of Cys-8E polymer. **b**^1^H NMR and **c** FTIR spectrogram of Cys-8E polymer. **d** Intracellular GSH of stem-like osteosarcoma cells treated with Cys-8E NP after 24h. **e** Appearance of NPs (left) and NP_J4+Apa_ (right). **f** NP_J4+Apa_ solution of DI water, PBS or 10% serum in DMEM solutions. **g** Fluorescence microscopy images of the SJSA-1 and 143B after incubation with free coumarin 6 (C6) or NP_C6_ for 30min (green, C6; blue, DAPI). Flow cytometric quantification of C6 fluorescence intensity after the treatment. The scale length of each image is 25 μm. **d** Data are expressed as mean ± SD. All results are from three independent experiments. P values were calculated by Student’s t test (***P < 0.001, **P < 0.01, or *P < 0.05).


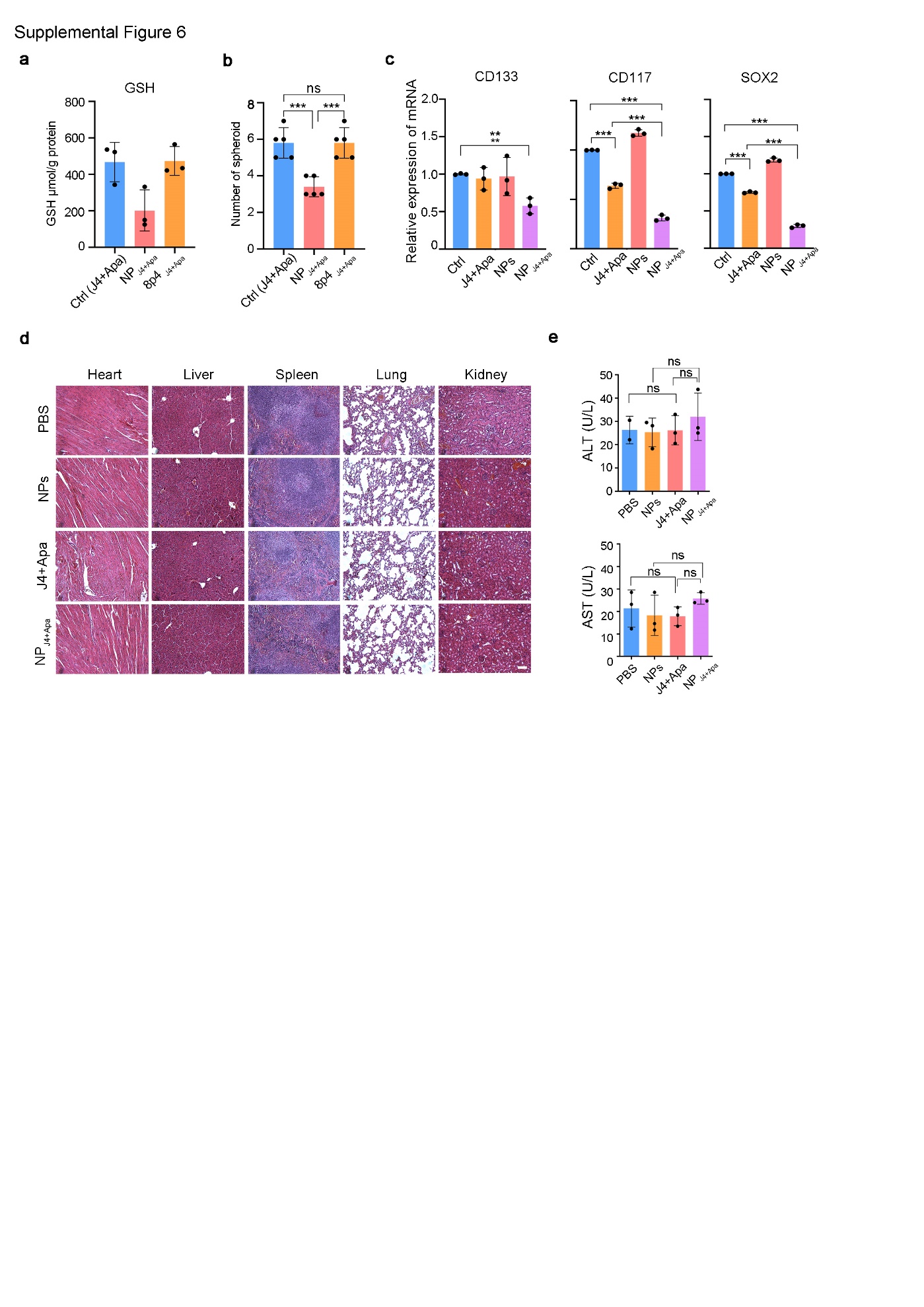


**Figure. S6.**

***In vivo* biosafety detection. Related to Figure 6. a** NP_J4+Apa_ showed GSH scavenging effect on 143B-derived tumor spheroids compared with free J4+Apa or 8P4 NP formulation with the J4+Apa loaded (8P4_J4+Apa_) but without GSH scavenging function. **b** The effects of NP_J4+Apa_ and 8P4_J4+Apa_ on sphere formation inhibition compared with free J4+Apa. **c** RT-PCR analysis shows mRNA levels of *CD133*, *CD117*, and *SOX2* were inhibited by NP_J4+Apa_ in 143B derived tumor spheroids. **d** H&E stained images of heart, liver, spleen, lung, and kidney in each mice group. The nude mice of the four groups were implanted with SJSA-1 cells and further treated by PBS, J4 and Apa, NPs, or NP _J4+Apa_ for 22 days at J4 3 mg/kg and Apa 6 mg/kg, respectively. Scale bar = 200 μm. **e** ALT and AST were measured after 24 days of observation from the serum of mice in all groups.

The data are expressed as mean ± SD. All results are from three independent experiments. P values were calculated by Student’s t test (***P < 0.001, **P < 0.01, or *P < 0.05).

**Table S1**

**Primers for RT-qPCR used in this study.**

| Gene | Forward | Reverse |
| --- | --- | --- |
| qPCR (Human) | |  |
| CHOP | GGAAACAGAGTGGTCATTCCC | CTGCTTGAGCCGTTCATTCTC |
| GADD34 | ATGATGGCATGTATGGTGAGC | AACCTTGCAGTGTCCTTATCAG |
| ATF4 | ATGACCGAAATGAGCTTCCTG | GCTGGAGAACCCATGAGGT |
| ATF3 | CCTCTGCGCTGGAATCAGTC | TTCTTTCTCGTCGCCTCTTTTT |
| ERN1 | CACAGTGACGCTTCCTGAAAC | GCCATCATTAGGATCTGGGAGA |
| TRIB3 | AAGCGGTTGGAGTTGGATGAC | CACGATCTGGAGCAGTAGGTG |
| CEBPB | CTTCAGCCCGTACCTGGAG | GGAGAGGAAGTCGTGGTGC |
| PMAIP | ACCAAGCCGGATTTGCGATT | ACTTGCACTTGTTCCTCGTGG |
| BAX | CCCGAGAGGTCTTTTTCCGAG | CCAGCCCATGATGGTTCTGAT |
| EZH2 | AATCAGAGTACATGCGACTGAGA | GCTGTATCCTTCGCTGTTTCC |
| FAS | AGATTGTGTGATGAAGGACATGG | TGTTGCTGGTGAGTGTGCATT |
